# Breastfeeding Behaviors and the Innate Immune System of Human Milk: Working Together to Protect Infants against Inflammation, HIV-1, and Other Infections

**DOI:** 10.3389/fimmu.2017.01631

**Published:** 2017-11-29

**Authors:** Bethany M. Henrick, Xiao-Dan Yao, Laila Nasser, Ava Roozrogousheh, Kenneth L. Rosenthal

**Affiliations:** ^1^Department of Food Science and Technology, University of California, Davis, Davis, CA, United States; ^2^Foods for Health Institute, University of California, Davis, Davis, CA, United States; ^3^McMaster Immunology Research Centre, Department of Pathology and Molecular Medicine, Michael G. DeGroote Institute for Infectious Disease Research, McMaster University, Hamilton, ON, Canada

**Keywords:** breast milk, breastfeeding behaviors, HIV-1, human breast milk stem cells, human milk oligosaccharides, innate immune bioactive factors, mother-to-child HIV transmission, soluble toll-like receptor 2

## Abstract

The majority of infants’ breastfeeding from their HIV-infected mothers do not acquire HIV-1 infection despite exposure to cell-free virus and cell-associated virus in HIV-infected breast milk. Paradoxically, exclusive breastfeeding regardless of the HIV status of the mother has led to a significant decrease in mother-to-child transmission (MTCT) compared with non-exclusive breastfeeding. Although it remains unclear how these HIV-exposed infants remain uninfected despite repeated and prolonged exposure to HIV-1, the low rate of transmission is suggestive of a multitude of protective, short-lived bioactive innate immune factors in breast milk. Indeed, recent studies of soluble factors in breast milk shed new light on mechanisms of neonatal HIV-1 protection. This review highlights the role and significance of innate immune factors in HIV-1 susceptibility and infection. Prevention of MTCT of HIV-1 is likely due to multiple factors, including innate immune factors such as lactoferrin and elafin among many others. In pursuing this field, our lab was the first to show that soluble toll-like receptor 2 (sTLR2) directly inhibits HIV infection, integration, and inflammation. More recently, we demonstrated that sTLR2 directly binds to selective HIV-1 proteins, including p17, gp41, and p24, leading to significantly reduced NFκB activation, interleukin-8 production, CCR5 expression, and HIV infection in a dose-dependent manner. Thus, a clearer understanding of soluble milk-derived innate factors with known antiviral functions may provide new therapeutic insights to reduce vertical HIV-1 transmission and will have important implications for protection against HIV-1 infection at other mucosal sites. Furthermore, innate bioactive factors identified in human milk may serve not only in protecting infants against infections and inflammation but also the elderly; thus, opening the door for novel innate immune therapeutics to protect newborns, infants, adults, and the elderly.

## Introduction

Although it had been recognized for centuries that breastfeeding and infant health were associated, one of the earliest systematic studies to demonstrate this was conducted by Grulee *et al*. in 1935 (1). They studied over 20,000 mother–infant dyads and showed that compared with breastfed infants, non-breastfed infants had 3.1-fold higher morbidity and 7.1-fold higher mortality due to gastrointestinal disease, 1.4-fold higher morbidity and 1.9-fold higher mortality due to respiratory disease, and 2.5-fold higher morbidity and 4.3-fold higher mortality from other diseases (1). These differences clearly indicate that breastfeeding and breast milk have protective activities. Indeed, it has been established that human milk contains a growing list of bioactive molecules, including components of the innate and adaptive immune system of the mother, primarily secretory immunoglobulin A (SIgA) (2–4). The title of this review is taken, in part, from Dr. David S. Newburg who coined the term “innate immune system of human milk” (2).

Newborns and infants bear the greatest burden of infectious disease. The World Health Organization estimates 10.6 million children under the age of 5 die every year with the highest mortality rates occurring in the first month of life (5, 6). Importantly, 95% of infant morbidity and mortality occur in low and middle-income countries, such as sub-Saharan Africa where HIV and TB are endemic. Universal breastfeeding could prevent annual deaths of 823,000 children under the age of 5 years as well as 20,000 breast cancer deaths each year (7). Economic losses close to three billion dollars per year are associated with not breastfeeding (8).

Breastfeeding unquestionably protects against death and disease. Studies conducted in low and middle-income countries have clearly demonstrated that exclusively breastfed infants are protected against morbidity and mortality with only 12% of the risk of death compared with those who were not breastfed (9). Non-breastfed infants younger than 6 months had 3.5 times (boys) and 4.1 times (girls) increased mortality compared with infants receiving breast milk (10). In high-income countries, breastfeeding has been shown to be associated with a 36% reduction in sudden infant death and a 58% decrease in necrotizing enterocolitis (11). Furthermore, breastfeeding protects infants against 50% of all diarrhea episodes and a third of respiratory infections in infants who are not breastfed (12).

These protective effects of breast milk undoubtedly can be attributed to the multitude of bioactive molecules that have been shown protective against infections, reducing inflammation, facilitating immune system and organ development, and beneficially influencing the infant microbiome. Since the majority of bioactive factors in milk have not yet been identified, characterization of novel factors in milk will open the door for the development of novel antimicrobial immunotherapeutics.

## Breastfeeding Behaviors and HIV-1

The benefits of breastfeeding for infants arise from the unique composition of breast milk. Breast milk completely nourishes the infant while establishing and promoting a healthy microbiome, and providing passive protection through maternal innate and adaptive immunological factors. Breastfeeding is well recognized to protect infants against gastrointestinal and respiratory infections, diarrheal diseases, and provides long-term health benefits (13–16). Additional socioeconomic benefits extend to the mother and family since breastfeeding promotes child spacing, social acceptance of the nursing woman, and is cost effective (17, 18).

It became clear in the 1980s that breast milk serves as a medium for HIV-1 transmission. The exact mechanism(s) of postnatal mother-to-child transmission (MTCT) of HIV remains unclear. In addition to *postnatal* transmission *via* breast milk, infants can become infected from their HIV-infected mothers *in utero* or following exposure to maternal fluids during parturition (19). However, this risk is significantly attenuated if the mother is given antiretroviral (ARV) therapy pre- and post-cesarean delivery (19). Without proper intervention strategies, an estimated 11–42% of infants will become infected from their HIV-infected mothers (19, 20) depending on factors such as maternal viral load and CD4 count, breast milk composition including innate immune factor levels, ARV treatment, breast pathology (particularly mastitis that can increase milk HIV RNA up to 10-fold in the affected breast), duration of breastfeeding, weaning practices and breastfeeding behavior (exclusive versus mixed) (21–24).

The past decade has shown significant progress in the prevention of mother-to-child transmission (PMTCT) of HIV globally through improved access to ARV therapies for women and infants, as well as the universal promotion of exclusive breastfeeding (EBF) when safe and sustainable alternatives are not readily available. As a result of these prevention strategies, for the first time the elimination of MTCT of HIV is considered a realistic public health goal (25). For example, ARV therapies such as single-dose nevirapine, given to the mother during delivery and the infant within 72 h postpartum has proven effective and has undoubtedly played an important role in the drastic decrease of approximately 800,000 cases of MTCT of HIV in 2002 to 300,000 cases in 2011 (26). Recently, an infant born infected with HIV was immediately treated with ARVs and cleared the infection (27). However, follow-up studies revealed that the HIV infection reappeared. Despite the effectiveness of these therapies, in 2011 only an estimated 57% of pregnant or lactating HIV-infected women globally were receiving any ARV therapy (25), largely due to the cost, lack of health-care support workers and inconsistent supply of ARVs (28). In many resource-limited areas where HIV-infected mothers have inadequate access to ARV therapies, mothers are encouraged to exclusively breastfeed their infants as an alternative preventive intervention (29).

Given that HIV-infected breast milk can contain high levels of cell-free virus (CFV) and cell-associated virus (CAV), it seems paradoxical that with prolonged and repeated exposure to HIV during EBF can significantly decrease postnatal MTCT of HIV compared with mixed feeding or non-exclusive breastfeeding (nEBF) (21–24). Thus, EBF infants of HIV-positive mothers who regularly consume HIV containing breast milk have increased protection against infection compared with mixed fed infants who are less frequently exposed to the virus. In four large cohort studies, EBF reduced HIV MTCT by 4- to 10-fold compared with nEBF or mixed feeding. Kuhn *et al*. (21) showed that nEBF more than doubled the risk of postnatal HIV transmission, while Iliff *et al*. (23) showed transmission rates as low as 1.3% in women who were EBF up to 6 months. Furthermore, there were no significant differences in viral load, CD4+ T cell levels or co-infections between the women who EBF and those that nEBF their infants that could account for the difference in transmission rates (22, 23). This preventative method is so effective in the PMTCT of HIV and in protection against enteric infections that the WHO recommends EBF despite the HIV status of the mother when safe and sustainable alternative feeding is unavailable (30). While the reasons underlying this phenomenon remain unanswered, they have been closely linked with innate immune factors in breast milk (31, 32). In addition, intestinal permeability is significantly increased during nEBF, replacement-fed (formula) and weaned infants which is associated with MTCT of HIV-1 (33). This strongly suggests that breast milk factors facilitate the nursing infant’s intestinal maturation and help maintain integrity of the intestinal barrier. We have hypothesized that short-lived innate factors in milk would have to be consistently provided to the nursing infant *via* EBF to sustain a protective innate immune threshold to prevent HIV transmission *via* milk. Although a number of comprehensive reviews of breastfeeding and HIV transmission have been published (34, 35), this article will highlight some of the current insights into biological and immunological factors in breast milk that are associated with protection from HIV infection *via* breastfeeding.

## Innate and Adaptive Immune Factors and Cells in Human Milk that Inhibit HIV

Humans are mammals because we have mammary glands which many believe evolved as part of the innate immune system (36). Innate and adaptive immune factors in breast milk have been shown to play critical evolutionary roles in protecting newborn infants against a wide variety of infections. Recently, of about 415 proteins identified in a pooled milk sample, 261 were novel (37). Importantly, many of these factors have not been well characterized, but many have immunomodulatory and/or antimicrobial activities critical to protecting the immunologically naïve infant and promoting intestinal development and microbiome. Consequently, identification of these novel factors in milk and elucidation of their functions could inform the development of novel therapeutics or vaccines.

## Non-Cellular Components in Human Milk

**Figure 1:**
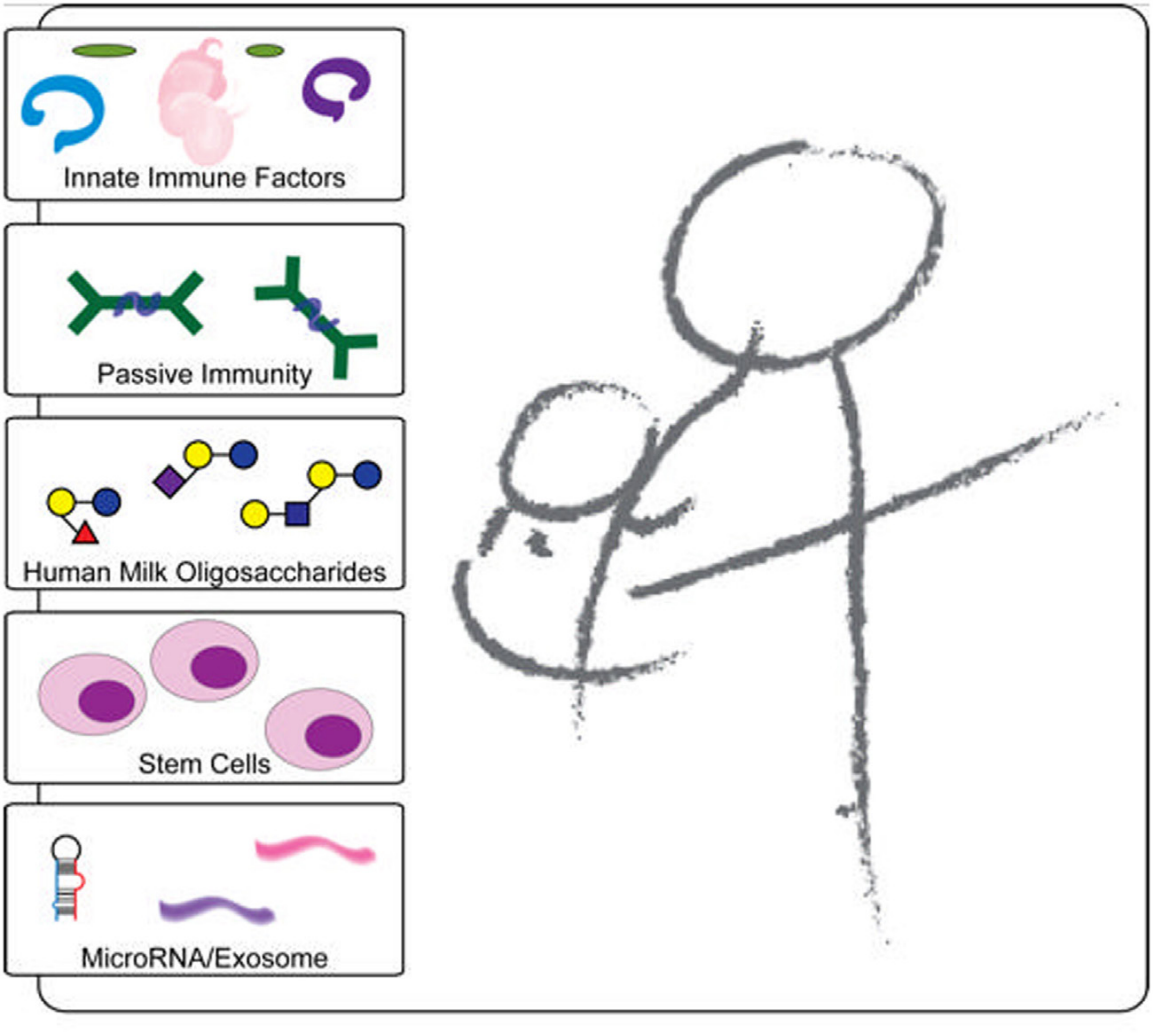
Schematic representation of the protective components in breast milk. Breast milk innate factors that have anti-inflammatory and antimicrobial properties are likely to affect the passage of HIV through the breastfeeding infant’s intestine by modulating the integrity of the intestinal mucosa and directly inactivating the virus. In contrast to exclusively breastfed infants, non-exclusively breastfed infants may be exposed to contaminated water and/or food antigens without a protective threshold level of innate factors in breast milk, which may lead to increased intestinal permeability and microbial translocation. In addition, the foreign antigens likely increase inflammation and recruit increased target cells leading to increased HIV transmission.

Non-cellular factors in breast milk that have been attributed to the protection from HIV infection observed in breastfed infants include innate factors, cytokines, and oligosaccharides (31, 32, 38) (Table 1). For other factors such as HIV-specific antibodies, the data are less clear (39, 40). The levels of many breast milk factors correlate with decreased MTCT of HIV and/or have direct anti-HIV activity *in vitro*, including lactoferrin, secretory leukocyte protease inhibitor (SLPI), mucin, and soluble toll-like receptor 2 (sTLR2) (41–44) (Figure 1). Specifically, lactoferrin, whose levels have been shown to correlate with reduced MTCT of HIV (45), has been shown *in vitro* to bind to the V3 loop of gp120, thus inhibiting gp120 interaction with host CD4 receptor (46). Lactoferrin has also been shown to inhibit bacterial-induced inflammation (47, 48). Similarly, SLPI levels correlated with decreased MTCT HIV transmission through breast milk (49), which is supported by *in vitro* studies indicating that it interacts with target cells to inhibit viral entry (50). Another component abundant in breast milk, mucin-1, can inhibit HIV infection *in vitro* by preventing dendritic cell (DC)-SIGN-mediated transmission of HIV from DCs to activated CD4+ T cells (41).

**Table 1:** Innate factors in human milk.

Lactoferrin	Inhibits gp120 interaction with CD4

Mucin-1	Prevents dendritic cell (DC)-SIGN mediated transmission from DCs to CD4+	Habte *et al*. (120, 121)

Secretory leukocyte protease inhibitor	Interacts with target cells	Farquhar *et al*. (49)

Tenascin-C	Neutralizes virions by binding to chemokine receptors on env	Fouda *et al*. (51)

Lysozyme	Inhibits HIV entry and replication	

Soluble toll-like receptor 2	Inhibits inflammation and HIV infection	Henrick *et al*. (44)

Human Milk Oligosaccharides	Pathogen blocking agents and decoy receptors	Newburg *et al*. (73)

Another recently identified innate factor in breast milk is Tenascin-C (TNC), which has the capability to neutralize HIV-1 virions by binding to the chemokine co-receptor binding site on the HIV-1 envelope (51). Like lactoferrin, TNC binds to an epitope on the V3 loop of the HIV-1 envelope protein, blocking the virus’s interaction with mucosal epithelial cells due to this electrostatic interaction (51). These investigators concluded that due to TNC’s broad spectrum HIV-neutralizing activity it may provide a prophylactic agent to be orally administered to infants before breastfeeding (51). TNC is also an extracellular matrix protein previously shown to be involved in wound healing and fetal brain development (52, 53). Unfortunately, it was recently shown that the amount of TNC correlated only weakly with overall innate HIV-neutralizing activity of breast milk of uninfected women, indicating that the amount of TNC in mucosal fluids was inadequate to impede HIV transmission (54). Further, polyclonal IgG from HIV-infected breast milk blocked neutralizing activity of TNC (54).

We were the first to demonstrate that breast milk sTLR2 directly interacts with specific HIV-1 structural proteins, namely, p17, p24, and gp41, thus inhibiting cellular activation and cell-free HIV infection *in vitro* (44, 55). Furthermore, sTLR2 has known antimicrobial properties that significantly inhibit pro-inflammatory cytokine production in human intestinal epithelial cells (IECs), as well as reducing bacterial-associated inflammation in mice without impairing microbial clearance (44, 55, 56), and therefore, if developed, may provide a novel prophylactic anti-inflammatory agent in breastfeeding infants (57).

Recently, trappin-2/elafin was shown to be a biomarker of resistance to HIV infection in cervico-vaginal lavage of highly exposed seronegative commercial sex workers in Kenya (58). Our lab went on to demonstrate that elafin is 130 times more potent against HIV compared with its precursor, trappin-2, and part of the antiviral activity of this antiprotease was due to modulation of innate sensing (59–61). Hence, it is anticipated that breast milk trappin-2/elafin along with other antiproteases might serve as natural host-based immunomodulatory molecules. Further, these factors function concomitantly to control aberrant microbial-induced inflammation, have direct antimicrobial effects, as was most recently shown by Pfaender *et al*. (62), and inhibit HIV–host interaction thus are protective against postnatal HIV transmission to infants.

Breast milk contains a range of cytokines, some of which could potentially influence immune function and directly correlate with MTCT HIV transmission. Specifically, the pro-inflammatory chemokine ligand 5 (RANTES/CCL5) indirectly inhibits HIV infection *in vitro* by binding to its ligand CCR5, thus inhibiting gp120 binding to its co-receptor which is integral to host–viral attachment (63). However, RANTES levels in breast milk positively correlated with increased MTCT of HIV (64). Conversely, levels of breast milk interleukin (IL) 15, a pleotropic cytokine involved in activating CD8+ T and NK cells, positively correlated with protection from MTCT of HIV (65). Furthermore, we showed a positive correlation of sTLR2 and IL-15 levels in breast milk (55), thus indicating that these protective factors can function in concert to reduce MTCT of HIV.

## Studies on African HIV-Exposed Infants and Breastfeeding

Over the past decade, more than two million HIV-exposed uninfected (HEU) infants were born each year. In sub-Saharan Africa, HEU infants suffer up to four times increased risk of dying in the first 2 years of life and increased risk for infectious morbidity (66). The etiology of increased susceptibility of HEU infants to infectious disease remains undetermined but is likely due to having their immune systems’ compromised.

A number of studies have been conducted on HEU infants relating breastfeeding to their health. One particular study conducted by John-Stewart *et al*. concluded that EBF of HIV-exposed infants decreased the likelihood of them falling ill to pneumonia (67). Specifically, of the 388 HEU infants in Kenya followed from birth to 12 months of age, the breastfed infants had a 47% lower chance of getting pneumonia with a 74% lower chance of being hospitalized due to pneumonia (67). It can be concluded from these studies that breastfeeding HEU infants has many benefits including lower risk of HIV infection, pneumonia, and infectious diseases.

In another study, Bork (68) discovered that HEU infants in Kesho Bora who were not breastfed had an increased risk of infection between birth and 2.9 months. HIV-infected pregnant women from five locations in Burkina Faso, Kenya, and South Africa involved in a study on MTCT in the Kesho Bora trail were asked to either exclusively breastfeed or formula feed their infants. In total, 751 infants were investigated for 2 years to see the health implications (particularly fever, diarrhea, vomiting, and other serious infections) in relation to feeding method. The results of this study further support that breastfeeding HEU infant’s has many positive implications on the health of the infants when compared with HEU infants who are not breastfed.

## Beneficial Effects of Human Milk Oligosaccharides (HMOs)

Human milk oligosaccharides consist of a family of structurally diverse, unconjugated sugars that are found at very high abundance (5–23 g/l) and high complexity in human milk. More than 200 different HMOs have been identified and are unique to human milk (69–71). Together, HMOs make up the third largest component of breast milk and are completely indigestible to infants. Although once believed to have no biological significance, it is now clear that they can function as pathogen blocking agents or decoy receptors for pathogenic microbes (72), have direct signaling ability (73, 74), and play a major role in establishing a healthy microbiome (75–78).

To cause intestinal infection, bacterial and viral pathogens often adhere to lectin–glycan structures on mucosal surfaces to initiate colonization. For example, a common cause of bacterial diarrhea and infant mortality is due to *Campylobacter jejuni*, which binds to α1,2-fucosylatd glycan. Although recently contested (79), previous manuscripts have shown that HMOs directly reduce microbial infections by serving as soluble decoy receptors, which prevent pathogen binding to these glycans (80, 81). Addition of soluble α1,2-fucosylated HMO blocks binding of *C. jejuni* to human intestinal mucosa and reduces colonization in mice (82). The beneficial effects of this HMO on reducing *C. jejuni*-associated diarrhea were confirmed in a prospective study on 100 mother–infant pairs in Mexico City (83). In addition, HMOs have antiviral properties. Most recently, specific HMOs, namely, 2′ and 3′ fucosylactose (2′FL and 3′FL, respectively) have been shown to structurally mimic histo-blood group aantigens, and thus block norovirus from binding to this surrogate (84). Interestingly, HIV gp120 envelope glycoprotein binds to DC-specific ICAM3-grabbing non-integrin (DC-SIGN) on human DCs, which is important for mother-to-child HIV transmission *via* breastfeeding. DC-SIGN, though, has higher affinity for the Lewis (Le) blood group antigens (85, 86). HMOs containing Le blood group antigens compete with gp120 for binding to DC-SIGN *in vitro* (87). In the breastfed infant, mucosal surfaces are covered with high levels of HMOs that may block HIV entry *via* DC-SIGN, which may contribute to the relatively inefficient MTCT of HIV *via* breastfeeding (88).

Human milk oligosaccharides may also directly modulate IEC and immune cell responses. *In vitro* studies of human ECs incubated with the HMO 3′-sialyllactose decreases expression of sialyltransferases resulting in reduced binding of enteropathogenic *Escherichia coli* to intestinal ECs (89–91) and can directly alter growth-related cell cycle gene expression in intestinal ECs (92). It has also been proposed that sialylated HMOs may affect T lymphocyte maturation and promote a more balanced Th1/Th2 cytokine response (93). Indeed, exposure to sialylated HMOs was shown to reduce IL-4 production in lymphocytes from adult patients with peanut allergy (94) and, more recently, ingestion of 2′FL and 6′sialyllactose was shown to reduce food allergy through induction of IL-10 (+) T regulatory cells and indirect stabilization of mast cells in an animal model (95). Together, these data suggest HMOs may contribute to allergy prevention.

Arguably most importantly, our group, as well as others, have extensively elucidated how oligosaccharides, originally identified as “bifidus factor” (96), help promote and establish a healthy microbiome of breast feeding infants. HMOs, as a complex mixture of free glycans, provide a perfect growth advantage to *Bifidobacterium infantis* (97), which has evolved to contain all glycosyl hydrolases necessary to utilize HMOs internally (98) resulting in beneficial short-chain fatty acids and other metabolite production. This favors the growth of commensals in addition to lowering luminal pH (99).

## Milk Components have Temporal and Spatial Specificity

There are various different protective factors in milk. Defense factors include maternal-derived SIgA and SIgM, oligosaccharides, anti-inflammatory factors, antioxidants, epithelial growth factors, and the aforementioned leukocytes and cytokines (100). One of the most prevalent antibodies present in breast milk, SIgA, is produced by plasma cells in the mammary gland (101). It is argued that SIgA B cells migrate from the pregnant mother’s gut to her breast before delivery, although migration of maternal IgA B cells is not well characterized. In mucosal tissues, SIgA is produced as a dimer in which two immunoglobulin monomers are linked by the J chain. Upward of 75,000 IgA-producing plasma cells are present in the normal human intestine with 3–4 g of IgA secreted daily. This significantly exceeds the production of all other immunoglobulin classes. Continuous production of large amounts of SIgA occurs in the absence of pathogen invasion and is driven by recognition of resident microbiota. IgA secreted in the gut lumen binds to mucus coating epithelial surfaces where it is involved in preventing attachment to the epithelium and invasion by pathogens. IgA can also neutralize microbial toxins, bacterial lipopolysaccharide, and viruses it encounters. The formation of IgA:antigen complexes can enhance the uptake and transcytosis of luminal antigen by M or microfold cells and facilitate its uptake by Peyer’s patch DCs (101).

Full-term neonates are deficient in SIgA especially at the colostral stage of lactation. Breastfeeding provides specific maternal-derived SIgA antibodies to protect the infant (102). Similarly, SIgM is also transferred *via* breast milk and is key in combating neonatal enteric antigens such as microorganisms and food proteins (13).

## Cellular Components

The biological relevance of breast milk cells in MTCT of HIV remains unclear. Indeed, there are arguments that infected cells both facilitate and protect against HIV transmission in breastfeeding infants (103–104). Depending on the stage of lactation, the predominant cells types in milk consist of various leukocytes, in colostrum (4 × 10^6^/ml) and mature breast milk (10^5^–10^6^/ml), and mammary epithelial cells (MECs). The majority of leukocytes in breast milk have an activated phenotype (105) and are comprised of macrophages (55–60%) and neutrophils (30–40%), while 5–10% are lymphocytes (~65% CD8+ T cells, 15% CD4+ T cells, and 20% B cells) (106–108).

## Stem Cells in Human Breast Milk

Although most studies of cells in breast milk have focused on leukocytes and their immunological activities, particularly postpartum, more recent exciting studies have identified pluripotent stem cells in breast milk. Using a well-known stem cell marker found in neural, bone marrow, pancreatic and epithelial stem cells, nestin (109–111), Cregan *et al*. were the first to identify human breast milk stem cells (hBSCs) in milk from full-term mothers (112). These dynamic cells, which account for an estim ated 10–15% of all breast milk cells, were later shown to be pluripotent and successfully differentiate breast milk stem cells into adipogenic, chondrogenic, and osteogenic lineages (113, 114). Moreover, some investigators hypothesize that these cells may promote growth and development of the infant (115). Indeed, in mouse models, ingested milk stem cells were shown to survive in the gastrointestinal tract (116, 117). Given these insightful studies, it is clear that additional investigation of hBSCs will not only further our understanding of how hBSCs ingestion in early life may reduce disease burden in later life but may also provide novel regenerative medicine to replenish and restore damaged tissues.

## Cell-Free Versus Cell-Associated HIV: Which is Responsible for MTCT of HIV?

Despite the increasing knowledge of breast milk virus, it remains unclear whether CFV or CAV is responsible for HIV-1 acquisition in the infant. Indeed, both CFV HIV RNA and CAV proviral DNA can be found in HIV-infected breast milk (when the mother is not receiving ARV therapy), and both levels correlate with postpartum MTCT of HIV (118, 119). Importantly, it has been shown that multiple innate immune factors that are endogenous to breast milk, including mucin, SLPI, sTLR2, lactoferrin, lysozyme, and oligosaccharides can effectively inactivate CFV infection *in vitro* (31, 32, 44, 55, 58, 88, 120–123), whereas innate immune factors seemingly have little to no effect on CAV infection *in vitro* (32, 124). CAV HIV-1 infection has been shown to be more efficient compared with CFV infection (125) and is significantly more difficult to neutralize (126), thus indicating that CAV might be responsible for postnatal MTCT transmission. Conversely, ARV therapy significantly decreases HIV RNA and correlates with reduction in breast milk transmission rates (127, 128), while proviral DNA levels remain largely unaltered (129, 130). These observations suggest that CFV likely plays an important role in breast milk transmission. Given these contradictory studies, it could be argued that multiple factors including overall maternal viral load, breast health (i.e., mastitis), innate immune factor levels, as well as feeding practices all contribute to the founding viral infection in the infant.

Based on a collection of studies examining cell-free and cell-associated HIV-1, there is a clear trend suggesting that CFV is more predominantly associated with viral load in the later stages postpartum. Ndirangu *et al*. showed that at 6 months, CFV was more heavily associated with HIV transmission. Up until 6 months, however, the viral load levels were analogous (131). Similarly, Koulinska *et al*. (130) showed that cell-free HIV-1 is a significant predictor of transmission after 9 months postpartum. In the earlier stages postpartum in both of the aforementioned studies, a 10-fold increase in both viral levels corresponded to a 3-fold increase in viral transmission (35, 131).

The first demonstration of selective transmission of HIV variants was conducted by Wolinsky *et al*. in 1992 (132) which showed that a minor subset of maternal virus was transmitted to the infant. However, the understanding of transmitted/founder viruses in breast milk is still not clearly defined (133, 134). Similar to other mucosally transmitted founder viruses, postnatal acquisition is primarily CCR5 tropic (134). Phylogenetic comparison of milk and plasma envelope gene sequences revealed that monotypic viruses are significantly more common in milk as compared with plasma from the same mother (133, 134), thus suggesting that the majority of breast milk viruses are produced by infected cells of the mammary gland. In addition, infant and maternal HIV variants did not differ in their sensitivity to broadly neutralizing antibodies; however, the viruses from transmitting mothers tended to be less sensitive to antibody-dependent neutralization (135). By contrast, other studies suggest that viral species found in the breast milk and plasma of infected mothers were genetically similar (136). Taken together, the role of breast milk cells in MTCT of HIV remains vague, and to explain these divergent observations, one possibility may be that the breast is continuously replenished with systemic CFV or CAV that can readily be transmitted to the breastfeeding infant and/or undergoes local replication in the mammary compartment (134). It is important to note that although MECs can endocytose cell-free HIV (137); whether or not the virus integrates into the host genome remains controversial (137, 138).

## Cells Involved in MTCT

The biological relevance of breast milk cells in MTCT of HIV also remains unclear. Indeed, there are arguments that infected cells both facilitate and protect against HIV-1 transmission in breastfeeding infants (103, 104, 139).

Macrophages and MECs are thought to facilitate MTCT of HIV-1 for multiple reasons. First, macrophages make up the majority of leukocytes in breast milk (106), are readily infected with HIV-1, and express DC-SIGN, a DC-specific receptor for HIV-1 that facilitates HIV-1 infection *in vitro* (103). In addition, oral administration of macrophages to newborn mice survived several hours and were found in the neonatal intestine (104). Second, MECs make up a substantial portion of the cells in breast milk (108). These cells express several canonical HIV receptors (including CD4 and CCR5), readily endocytose cell-free HIV-1, and can act as a viral reservoir (137, 138). Furthermore, our laboratory recently found that MECs exposed to cell-free R5 HIV-1 on the basolateral surface readily transcytosed virus through the monolayer without damaging tight junctions but did not integrate virus into the genome (data not shown). Moreover, HIV-1 basolateral exposure significantly altered TLR expression, led to significantly elevated pro-inflammatory cytokine production in both apical and basolateral compartments, and increased CCR5 expression. Viral production in MECs, CD4+ cells, and breast milk macrophages has previously been shown (103, 138, 140). In fact, the HIV-infected CD4+ T cells in breast milk are 17 times more effective than in blood (35).

Multiple target sites in the nursing infant’s gastrointestinal tract, including oral, esophageal, and intestinal mucosal epithelium have been proposed. Specifically, the oral epithelium has been shown to be permissive to both CFV and CAV *in vitro* (141). Yet, the oral environment also has effective anti-HIV properties (31) making MTCT HIV transmission possible, albeit at a very low incidence (141). A recently published *in vivo* infection model demonstrated that humanized mice were readily infected with HIV through the oral cavity; however, breast milk had strong inhibitory effects on both CFV and CAV (142). A persuasive argument has been made for MTCT HIV transmission through the infant’s intestine through multiple studies showing that mixed feeding doubled the risk of an infant acquiring disease compared with EBF (21–23, 143). In addition, a previous publication reported significantly increased lipopolysaccharide (LPS) levels in infants that were nEBF or weaned, indicating a disruption in the intestinal mucosa (33). Notably, this seminal publication showed that systemic LPS levels were a significant predictor of MTCT through breast milk, thus indicating that tight junctions were disrupted in the infant’s intestinal mucosa. Previously, Nazli *et al*. (144) showed that HIV-dependent production of pro-inflammatory cytokines disrupted tight junctions in IECs.

## Next Steps in Research on Innate Immune Factors: sTLR2

We showed that sTLR2 inhibited bacterial and viral-induced cellular activation in intestinal cells (44, 55, 57) and HIV virions induced cellular activation through a TLR-mediated pathway leading to increased infection *in vitro* (145). This indicates that sTLR2 likely plays a role in inhibiting HIV and/or bacterial-induced cellular activation directly at the infant’s intestinal mucosa.

Toll-like receptors are evolutionarily conserved transmembrane pattern recognition receptors (PRRs) that recognize highly conserved pathogen-associated molecular patterns (PAMPs). They are part of the first line of defense against pathogen invasion and trigger innate immune responses and subsequent antigen-specific adaptive immunity. The 10 TLRs in humans recognize highly conserved molecules broadly shared and also expressed by pathogens, but not found in mammals, such as dsRNA, ssRNA, flagellin, CpG DNA, and LPS either intracellularly or extracellularly.

Historically, TLRs have not been extensively evaluated for their role in MTCT of HIV, despite the fact that soluble TLRs provide the most direct attenuation of inflammation and innate immune responses to pathogens by binding PAMPs, thus effectively inhibiting PAMP–PRR engagement (146). LeBouder *et al*. (147) were the first to identify forms of sTLR2 in breast milk and plasma and through computational molecular docking revealed a cylindrical arrangement between sTLR2 and soluble CD14 that encapsulates synthetic bacterial lipoprotein Pam_3_CSK_4_ preventing bacterial-induced cellular activation through membrane-bound TLR2. Additional publications have highlighted sTLR2’s role in significantly inhibiting bacterially induced pro-inflammatory cytokine production *in vitro* in oral epithelial cells, placental tissue explants, and human IECs (44, 55, 148, 149).

Although sTLR2 is important in regulating bacteria-induced cellular activation, sTLR2-dependent regulation of immune activation during viral infection remains poorly understood. Accruing evidence indicates that a range of soluble molecules, including defensins, interferons, antiproteases, and chemokines suppress and control viral infections (59, 150). In addition, our investigation showed that sTLR2 directly interacts with HIV PAMPs, including p17, p24, and gp41, which leads to significantly reduced IL-8 production, CCR5 expression, NFκB activation, and HIV infection in a dose-dependent manner (55).

In proceeding with this research to solidify the evidence, the next step is to establish the mechanism by which this interaction takes place. In support of this hypothesis, we also showed that sTLR2 has a very short half-life at physiological temperatures (44). Interestingly, sTLR2 levels were significantly increased in HIV-infected compared with uninfected breast milk samples, and significantly correlated with p24 [a marker of viremia (151)] and IL-15 concentrations (55). The correlation between sTLR2 and IL-15 might have important implications since IL-15 in breast milk has been shown to correlate with decreased MTCT of HIV (65). These findings not only have important implications for our fundamental understanding of HIV infection and pathogenesis but also have the potential to inform novel therapeutic approaches to prevent mucosal transmission of HIV, including MTCT. We envision that sTLR2 alone or combined with other innate antiviral factors, e.g., IL-15, could be directly added to expressed breast milk and orally administered to infants to augment prevention of HIV mucosal transmission. This would, however, be limited to addition to breast milk, as sTLR2 would not be stable in acidic environments such as the gastrointestinal tract and vaginal mucosa according to our observations. Importantly, host innate factors, including soluble TLR immunotherapeutics, are unlikely to be toxic. Similarly, sTLR2, alone or in combination with other innate factors, might be useful as a natural immunomodulatory molecule to prevent sexual transmission of HIV-1.

## Breast Milk, MicroRNAs (miRNAs) and Exosomes: Regulators of Growth, Development, and Immune Protection

Human milk contains short, non-coding single RNA molecules called miRNAs that are approximately 22 nucleotides in length. Compared with other body fluids, milk is one of the richest sources of miRNAs, which are present in all three fractions of human milk, including cells, lipids, and skim milk (152). miRNAs serve as key regulators of gene expression at the posttranscriptional level by binding to an mRNA target to either inhibit translation of mRNA into protein or promote its degradation (153–155). miRNAs are first transcribed into primary microRNA (pri-microRNA) from specific genes on DNA by RNA polymerase II, and then are converted into hairpin precursor microRNA (pre-microRNA) by the Drosha–DGCR8 complex. The enzyme Dicer then produces mature miRNA from pre-microRNA in the cytoplasm (156, 157). A single mature miRNA can bind and regulate multiple mRNAs (158). Studies indicate that miRNAs can regulate up to 50% of protein synthesis (154).

Importantly, miRNAs play a key role in regulating the immune system, including T and B cell development (159, 160), release of inflammatory mediators (161), proliferation of monocytes and neutrophils (162), and differentiation of macrophages and DCs (163).

Breast milk miRNAs are frequently packaged in vesicles such as exosomes which play an important role in their survival under harsh conditions (164, 165). Indeed, since the nursing infant’s gut is less acidic and more permeable compared with adults, this provides strong support for survival, adsorption, and integration of milk miRNAs in the infant and facilitate early growth, protection, and development. Thus, it is important to characterize miRNAs in human milk and examine factors that might influence it.

Of 12 body fluids examined, breast milk contained vastly more miRNAs than any other fluid tested, including greater than 80-fold the concentration found in amniotic fluid and colostrum (152). miRNAs are resistant to acidic conditions, digestion by RNAse, incubation at room temperature and various freeze thaw cycles (166–169). In breast milk, this resistance is due to the fact that miRNAs are contained in exosomes or microvesicles. Treatment with detergent, Triton-X, that disrupts lipid membranes, results in degradation of miRNAs by RNAse (170). Resistance to acidic conditions ensures passage through the stomach and adsorption into the bloodstream, which in turn allows the exchange of genetic information between mother and offspring. Valadi *et al*. were the first to demonstrate that exosome-mediated transfer of mRNAs and miRNAs is a novel mechanism of genetic exchange between cells (171). Secreted miRNAs represent a newly recognized layer of gene regulation and intercellular communication (172–174), while exosomal miRNAs play a pivotal role in horizontal miRNA transfer (174) as was originally shown by Raposo *et al*. who provided the first evidence for exosome-mediated immune cell communication (175).

MicroRNAs were shown to play a critical role in innate antiviral defense (176–178). This was demonstrated by Triboulet *et al*. who knocked down two important miRNA processing proteins, Drosha and Dicer, resulting in significant enhancement of viral replication in peripheral blood mononuclear cells (PBMCs) from HIV-infected patients and in latently infected cells (179). Interestingly, they also demonstrated that knockdown of some of these effectors led to virus reactivation in PBMCs isolated from HIV-infected patients undergoing suppressive combination ART (180). These studies highlight the importance of miRNs in modulating HIV-1 infection. In a ground-breaking study, Huang *et al*. showed that a selected group of miRNAs, including miR-28, miR-125b, miR-150, miR223, and miR-382, bound to the 3′ UTR of viral mRNAs and showed that activation of resting CD4+ T cells resulted in downregulation of these miRNAs, which correlated with enhanced HIV-1 susceptibility (181). Further, experiments in which all five of these miRNAs were inhibited in resting CD4+ T cells from cART-treated individuals (with undetectable viremia) displayed enhanced HIV-1 production indicating that “anti-HIV-1 miRNAs” contribute to HIV-1 latency in resting CD4+ T cells. Thus, these studies suggest these novel anti-HIV-1 miRNAs could play a role in controlling HIV latency and their manipulation could potentially contribute to purging of viral reservoirs (181).

Other modulators of anti-HIV-1 miRNAs include cytokines and TLR ligands. For example, stimulation of TLR3 was shown to induce an anti-HIV effect in primary macrophages, partially through upregulation of miR-28, miR-125b, miR-150, and miR-382 (182). More recently, activation of TLR3 in primary human macrophages resulted in significantly enhanced expression of miR-155 that correlated with decreased HIV-1 infectivity (183). These investigators also showed that miR-155 inhibited HIV-1 at a postentry, pre-integration step (183). Together, these findings indicate interplay between miRNAs, TLRs, and HIV-1 that have important implications for HIV-1 infection, replication, and chronic immune activation (184).

## Conclusion and Future Directions

The majority of infants’ breastfeeding from their HIV-infected mothers do not acquire HIV. Indeed, EBF has been one of the most successful interventions in protecting infants in resource-limited countries from a broad range of infectious diseases, including HIV. Although the reason for this remains unclear, coordination of a number of innate immune factors in breast milk seem crucial for providing protection when infants are most vulnerable. Identification and characterization of natural immune factors that protect susceptible individuals from acquiring HIV might facilitate the production of novel innate immunotherapeutics in the near future. Given that a number of innate factors have demonstrated anti-HIV activity, and ensuing decreased MTCT, it can be concluded that innate factors are indeed a viable option to pursue as protective therapies. In addition, other factors such as sTLR2 and IL-15 have shown promising results and should be pursued to further understand their mechanisms of binding and blocking HIV-1 MTCT.

Human milk is a gold mine of uncharacterized, natural innate bioactive factors that have great promise to be developed and utilized for therapy or treatment of infections and inflammatory conditions in infants as well as adults and the elderly.

## Author Contributions

KR initiated the review article, contributed to the design and conductance of the study, collection of relevant literature, and writing and editing of the manuscript. BH played a key role in design and conductance of the study. She performed experiments noted in the review, analysis of the data as well as contributing to the writing and editing of the review. Similarly, X-DY carried out much of the experimentation and bench research. She also made major contributions to analysis of the data and writing of the manuscript. Both BH and X-DY spent substantial time working on our projects in Kenya and South Africa. LN and AR were students who contributed to collecting, reading, and identifying relevant literature for the review. All authors contributed to editing the review. LN is now a resident physician at McMaster University Health Sciences Centre.

## Conflict of Interest Statement

The authors declare that the research was conducted in the absence of any commercial or financial relationships that could be construed as a potential conflict of interest.
